# Editorial: Editors’ showcase: mental health occupational therapy

**DOI:** 10.3389/fpsyt.2025.1664227

**Published:** 2025-08-14

**Authors:** Ellie Fossey, Justin Newton Scanlan

**Affiliations:** ^1^ Department of Occupational Therapy, Monash University, Frankston, VIC, Australia; ^2^ Sydney School of Health Sciences, The University of Sydney, Sydney, NSW, Australia

**Keywords:** return to work, work environment, time use patterns, social participation, mental health recovery, recovery colleges, self-management, occupational therapy

We are very proud to introduce the first Research Topic to be presented in the Mental Health Occupational Therapy section of Frontiers in Psychiatry. The Mental Health Occupational Therapy journal section is committed to advancing knowledge about how occupational therapy practitioners (alongside people with lived and living experience of mental health or emotional challenges, families and carers, and colleagues from other disciplines) contribute to supporting the mental health recovery and wellbeing of individuals, families and populations in diverse cultural and service contexts globally.

Just over 10 years ago, we wrote an Editorial together encouraging mental health occupational therapy practitioners and researchers to expand the focus of their work to reflect the breadth of scope of occupational therapy practice (1). Reflecting on progress since then, we are pleased to see such a diversity of papers presented in this first Research Topic, the Editors’ Showcase: Mental Health Occupational Therapy. It really reflects many of the ways in which occupational therapy can support people with diverse challenges to work towards optimal mental health and wellbeing.

The nine papers represent contributions to understanding how participation in occupations of daily life and mental health interrelate, and to how occupational therapists promote mental health recovery and wellbeing. The contributors include international and interdisciplinary collaborations and they report research undertaken with wide-ranging populations and in diverse contexts, including workplaces, primary care and community mental health services. Broadly, key themes focus on the impact of work conditions on mental health and support for return to work; approaches for exploring and enabling self-determined, meaningful and rewarding forms of activity engagement; and training for recovery oriented practice. The individual papers address:

- The work experiences of recent immigrants in precarious work environments and how work conditions can impact their mental health and wellbeing, highlighting initiatives necessary to create safe and culturally friendly workplaces (Shankar et al.)

- Occupational therapy practitioners’ involvement with public safety personnel who experienced psychological injury during the return to work process, which suggests more timely pathways may influence return to work outcomes (Edgelow and Fecicia)

- An innovative occupational therapy practitioner-led program in primary care, which aims to enhance interprofessional collaboration and to promote recovery and sustainable return to work outcomes for employees on sick leave for common mental disorders (Labouret et al.)

- The importance of exploring and supporting meaningful time use in the context of community mental health services (Fossey et al.)

- The patterns of participation in daily life activities among individuals living with Post Traumatic Stress Disorder and a reflection on how occupational therapy may support more personally meaningful and rewarding participation (Shapira et al.)

- The relationships between symptom impact, cognition, sensory modulation and patterns of participation reported by individuals with recent-onset mental illness in inpatient and community programs, and suggestions regarding possible areas for early intervention (Lipskaya-Velikovsky et al.)

- The identification of factors influencing social participation post discharge from hospital among individuals living with mental illness who received occupational therapy support in acute care (Nagashima et al.)

- An innovative application of a well-established occupational therapy approach more commonly used in pediatrics (Cognitive Orientation to daily Occupational Performance [COOP]), which shows promise as a way of supporting individuals experiencing high-prevalence mental disorders to develop personalized strategies that address complex needs and enhance their engagement in meaningful activities (Wong et al.)

- Mental health practitioners’ experiences of Recovery College courses and their benefits, which highlights the potential of Recovery Colleges to offer enriching and accessible training to develop capabilities for recovery - oriented practice among mental health practitioners, including occupational therapy practitioners (Bellemare et al.)

We hope that occupational therapy practitioners and others across the world engage with this diverse array of research articles and use this knowledge to not only improve occupational therapy practices but also to encourage further research that advances the evidence informing occupation focused approaches for supporting the mental health and wellbeing of all individuals and enabling communities to thrive.
